# Photovoltaic Power Injection Control Based on a Virtual Synchronous Machine Strategy

**DOI:** 10.3390/s24134039

**Published:** 2024-06-21

**Authors:** Miguel Albornoz, Jaime Rohten, José Espinoza, Jorge Varela, Daniel Sbarbaro, Yandi Gallego

**Affiliations:** 1 Department of Electrical Engineering, Universidad de Concepción, Concepción 4070386, Chile; miguelalbornoz@udec.cl (M.A.); jose.espinoza@udec.cl (J.E.); jorgevarela@udec.cl (J.V.); dsbarbar@udec.cl (D.S.); 2 Department of Electrical and Electronic Engineering, Universidad del Bío-Bío, Concepción 4051381, Chile; ygallego@ubiobio.cl

**Keywords:** virtual synchronous machine, synchronverter, PV power injection

## Abstract

The increasing participation of photovoltaic sources in power grids presents the challenge of enhancing power quality, which is affected by the intrinsic characteristics of these sources, such as variability and lack of inertia. This power quality degradation mainly generates variations in both voltage magnitude and frequency, which are more pronounced in microgrids. In fact, the magnitude problem is particularly present in the distribution systems, where photovoltaic sources are spread along the grid. Due to the power converter’s lack of inertia, frequency problems can be seen throughout the network. Grid-forming control strategies in photovoltaic systems have been proposed to address these problems, although most proposed solutions involve either a direct voltage source or energy storage systems, thereby increasing costs. In this paper, a photovoltaic injection system is designed with a virtual synchronous machine control strategy to provide voltage and frequency support to the grid. The maximum power point tracking algorithm is adapted to provide the direct voltage reference and inject active power according to the droop frequency control. The control strategy is validated through simulations and key experimental setup tests. The results demonstrate that it is possible to inject photovoltaic power and provide voltage and frequency support.

## 1. Introduction

The decarbonization goals of power grids worldwide have led to increased participation of renewable energy sources, particularly solar photovoltaics (PV), which are cheap and scalable due to the modularity of PV modules. This energy source is present in microgrids, distribution grids, and large power systems [1]. Recently, grids with significant PV participation have experienced an increase in power quality issues; specifically, problems related to voltage magnitude at multiple buses in distribution grids and lack of inertia [2,3,4]. Regarding the first issue in [5], an analysis of the voltage magnitude profile of a grid was conducted, where it was observed that several buses presented overvoltage during peak irradiance hours. The explanation for this is that in distribution systems, the resistance of the power lines is not negligible compared to the reactance, preventing the decoupling between active and reactive power with respect to voltage magnitude, as well as the fact that in peak irradiance hours the demand is not at the peak. In [6], an analysis of the hosting capacity is made, considering different power quality issues related to PV power generation; the authors concluded that voltage magnitude problems are some of the main issues limiting PV penetration in power grids. Regarding the frequency fluctuation in power grids—this is due to the lack of inertia of power converters. Since these devices do not have a rotating mass, they lack a considerable energy reserve to compensate for imbalances between generation and demand [4].

According to [5,7,8,9,10], traditional voltage mitigation techniques, such as capacitor banks or transformers with tap changers, are not sufficient to compensate for voltage fluctuations in a high PV penetration environment because these solutions were not designed to operate in grids with bidirectional power flows or abrupt power changes. Some solutions that could improve power quality are battery energy storage systems, smart load control, PV curtailment, reactive power control strategies applied to PV inverters [5,7,11], and reactive power management of distributed generators, where photovoltaic sources have high reactive power availability, as demonstrated in [12,13], where the authors apply a distributed reactive power management algorithm in a multi-agent distribution system, achieving an improved voltage profile. Although a distributed power management system presents good results, the difficulty of implementation is that it depends on whether the distribution network has end users capable of providing compensation services and whether there is a system of economic transfers that promotes the ability of end users to cooperate [14].

An alternative that allows for the control of reactive power flows and the stabilization of voltage magnitude in grid buses is a flexible alternating current (ASC) transmission system (FACTS), which consists of power converters specialized in providing compensation functions. In [15], the authors conducted a study on the voltage compensation capabilities of different FACTS devices through reactive power injection. The results showed that the static synchronous compensator (STATCOM) is one of the best devices for compensating voltage and improving the power capacity of a transmission line. Recently, taking advantage of the similarity of the power converter topology, researchers proposed integrating STATCOM functions into PV inverters to provide voltage support in addition to PV power injection. These voltage support functions could be fully or partially implemented depending on the operating region of the power converter and the severity of the power quality problem [16,17,18].

The aforementioned technology can compensate for reactive power and provide voltage support, but not frequency support. For frequency compensation, inertia emulation using batteries has shown good results. In [19], the case of a small electrical network was studied, where inertia emulation using a battery system managed to reduce frequency drops and stabilize the frequency against disturbances in low inertia scenarios. On the other hand, the great disadvantage of this solution involves the costs associated with the energy storage system. Another solution to this problem involves grid-forming control strategies—some of which are based on a virtual synchronous machine, which consists of controlling an inverter to mimic the behavior of real synchronous machines. The advantage of this is that the inverter obtains the capabilities of a synchronous machine, such as frequency support through droop control, inertia, auto-tuning between inverters, voltage droop control, etc. [20]. A variant of this type of strategy is known as the synchronverter [21], which is based on the model of a two-pole wound rotor synchronous machine without considering magnetic saturation or damping windings. The synchronverter is capable of doubling the synchronous machine, although its implementation involves using a direct current (DC) constant voltage source.

Many studies have been carried out on stability, synchronization methods, dynamic behavior improvement, and decoupling of the synchronverter active and reactive power loops [22,23,24], but in all these papers, the DC source is considered a constant source with important energy reserves, which behaves differently from a solar panel array. In [25], the simulations of a grid and a synchronverter with a PV array as the DC source were studied, and the results showed that the PV power injection was enhanced using the synchronverter. However, the synchronverter did not show the ability to adjust its active power injection to frequency variations in the stationary state. On the other hand, in [26], the authors applied the virtual synchronous machine strategy with a phasor-based generator model in a two-stage PV generator to mitigate power oscillations. Among the results, the findings emphasize the strategy’s ability to suppress frequency oscillations faster with greater participation from this type of generator; moreover, the need to maintain solar energy reserves to provide support when there is an abrupt increase in demand is highlighted.

In this paper, we propose implementing a synchronverter-type control in a photovoltaic inverter to provide voltage and frequency support to the electrical system. Unlike the work mentioned above, it is intended to apply frequency support without considering a constant DC source or energy storage as batteries. Due to the inherent relationship between the power reserves of the electrical system with the frequency, it is necessary to adjust the power injection from the photovoltaic arrangement, i.e., to decrease the injected power when the frequency increases or to increase the power injection when the network frequency decreases. Regarding the latter, it is necessary to operate the PV array at a point other than the maximum power. The advantage of this method is that the regulation can be achieved without additional components such as batteries or other storage systems. The disadvantage lies in the economic losses incurred by not making the most of the available energy, the impact of which will be studied in future work.

Section 2 presents the equations of the proposed system, Section 3 is a small signal analysis of the synchronverter. Section 4 focuses on the maximum power point tracking (MPPT) used in this work and the modifications made to obtain new DC voltage reference values. Finally, the simulation and experimental results are presented in Section 5.

## 2. System Equations

**Figure 1:**
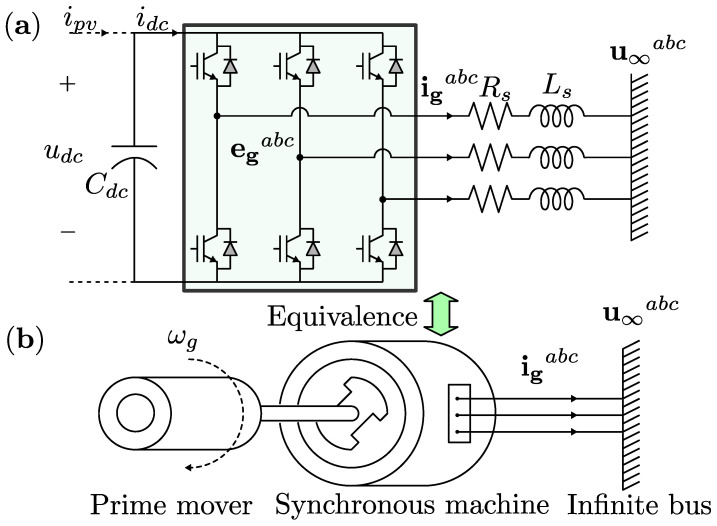
(**a**) Inverter and (**b**) synchronous machine connected to the infinite bus.

There is a similarity between the stator circuit of a synchronous machine and the AC side circuit of the inverter, as shown in Figure 1. Therefore, the electrical power system equations are the same for both systems. The mechanical equations of the synchronous machine are implemented in the microcontroller of the inverter to digitally twin the behavior of the first one. Therefore, this kind of control strategy is called a virtual synchronous machine.

### 2.1. Power System Equations

The equations of the power circuit describe the dynamics of the AC and DC voltages. These equations are obtained from the Kirchhoff voltage and current law. Considering a rotatory frame, these equations are given by the following:

(1a) $$\begin{array}{cc} \frac{di_{g}^{d}}{dt} & =-\frac{R_{s}}{L_{s}}i_{g}^{d}+\omega i_{g}^{q}+\frac{1}{L_{s}}e_{g}^{d}-\frac{u_{\infty }^{d}}{L_{s}} \end{array}$$

(1b) $$\begin{array}{cc} \frac{di_{g}^{q}}{dt} & =-\frac{R_{s}}{L_{s}}i_{g}^{q}-\omega i_{g}^{d}+\frac{1}{L_{s}}e_{g}^{q}-\frac{u_{\infty }^{q}}{L_{s}} \end{array}$$

(1c) $$\begin{array}{cc} \frac{du_{dc}}{dt} & =\frac{1}{C_{dc}}i_{pv}-\frac{1}{C_{dc}}\left(m^{d}i_{g}^{d}+m^{q}i_{g}^{q}\right) \end{array}$$

where the *d* and *q* super indexes indicate direct and quadrature axes, respectively, $i_{g}$ denotes the grid current, $e_{g}$ denotes the phase output voltage, $u_{\infty }$ denotes the infinite bus voltage, ω denotes the angular frequency used in the Park transformation, *m* denotes the modulator signal, $R_{s}$ denotes the resistor, $L_{s}$ denotes the inductance of the inductive filter, $C_{dc}$ denotes the capacitance, and $i_{pv}$ denotes the DC input current.

### 2.2. Synchronverter Equations

The dynamic equations of the rotor are as follows:

(2) $$\frac{d\omega_{g}}{dt}=\frac{1}{J_{g}}\left[T_{m}-T_{em}+D_{p}\left(\omega_{n}-\omega_{g}\right)\right]$$

where $\omega_{g}$ denotes the rotor angular speed, $J_{g}$ denotes the inertia of the rotor, $T_{m}$ denotes the input torque, $T_{em}$ denotes the electromechanical torque, $D_{p}$ denotes the torque droop coefficient, and $\omega_{n}$ denotes the nominal rotor angular speed.

(3) $$\frac{d\delta }{dt}=\omega_{g}-\omega_{\infty }$$

where δ denotes the power angle and $\omega_{\infty }$ denotes the angular frequency of the infinite bus bar. The input torque is calculated by the following:

(4) $$T_{m}=\frac{P_{g}^{*}}{\omega_{n}}$$

where $P_{g}^{*}$ denotes the active power reference. The voltage ${e_{g}}^{dq}$ is given by the following:

(5) $${e_{g}}^{dq}=\left[\begin{array}{c} e_{g}^{d}\\ e_{g}^{q} \end{array}\right]=\frac{3\omega_{g}\lambda_{f}}{2}\left[\begin{array}{c} \cos\;\delta \\ \sin\;\delta \end{array}\right]$$

where $\lambda_{f}$ denotes the magnetic flux. Considering Equation (5), the electromechanical torque is defined by the following:

(6) $$T_{em}=\frac{P_{g}}{\omega_{g}}=\frac{\left(e_{g}^{d}i_{g}^{d}+e_{g}^{q}i_{g}^{q}\right)}{\omega_{g}}=\frac{3\lambda_{f}\left(i_{g}^{d}\cos\;\delta +i_{g}^{q}\sin\;\delta \right)}{2}$$

where $P_{g}$ denotes the active power injected. The magnetic flux is given by the following:

(7) $$\frac{d\lambda_{f}}{dt}=\frac{1}{K_{q}}\left(Q_{g}^{*}-Q_{g}\right)+\frac{D_{q}}{K_{q}}\left(\sqrt{\frac{2}{3}}U_{n}-u_{\infty }^{d}\right)$$

where $U_{n}$ denotes the nominal line voltage, $Q_{g}^{*}$ denotes the reference of reactive power, $Q_{g}$ denotes the reactive power injected, $K_{q}$ denotes the positive constant, and $D_{q}$ denotes the reactive power droop coefficient. The inverter is controlled by the modulating signals; therefore, it is necessary to define these signals in terms of the virtual synchronous machine variables. These modulators are defined in a similar way as in [24], although in this case, both components d and q are considered:

(8) $$m^{dq}=\left[\begin{array}{c} m^{d}\\ m^{q} \end{array}\right]=\frac{2}{u_{dc}}\left[\begin{array}{c} e_{g}^{d}\\ e_{g}^{q} \end{array}\right]=\frac{2\omega_{g}\lambda_{f}}{u_{dc}}\left[\begin{array}{c} \cos\;\delta \\ \sin\;\delta \end{array}\right]$$

The parameters of (2) and (7) are adjustable, so according to [24], these parameters are calculated using the following equations:

(9) $$\begin{array}{ccccc} D_{p}=\frac{S_{n}}{\Delta \omega \cdot \omega_{n}^{2}}, & \; & D_{q}=\frac{S_{n}}{\Delta U\cdot U_{n}}, & \; & J_{g}=\frac{2S_{n}H}{\omega_{n}^{2}} \end{array}$$

where $S_{n}$ denotes the apparent nominal power, Δω and ΔU are the deviations of frequency and voltage, respectively, which are necessary to increase the input power from zero to nominal power, and *H* denotes the inertia time constant.

To synchronize the digital controller’s internal variables with the voltage oscillation, a phase-locked loop (PLL) is used in this paper, as described in [27].

### 2.3. DC Voltage Control Loop

The power injection control of a photovoltaic array is achieved through a DC voltage control loop. The voltage is determined by the energy stored in the capacitor, defined by the following:

(10) $$P_{pv}=\frac{1}{2}C_{dc}\frac{du_{dc}^{2}}{dt}+P_{g}$$

where $P_{pv}$ denotes the power injected by the PV array. Considering that the output of the control loop is the square of the DC voltage and the input is the active power supplied to the grid, the transfer function of (10) is as follows:

(11) $$G_{u_{dc}}\left(s\right)=\frac{U_{dc}^{2}\left(s\right)}{P_{g}\left(s\right)}=-\frac{2}{C_{dc}s}$$

where $G_{u_{dc}}\left(s\right)$ denotes the transfer function of the DC voltage capacitor.

**Figure 2:**
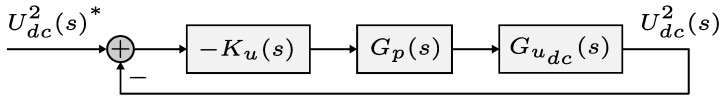
DC voltage control loop.

Figure 2 shows the DC voltage control loop, where $G_{p}\left(s\right)$ denotes the inner loop transfer function. Considering that the dynamics of the inner loop are 10 times faster than those of the DC voltage loop, $G_{p}\approx 1$, $U_{dc}^{2}\left(s\right)$ is the DC voltage square and $U_{dc}^{2}{\left(s\right)}^{*}$ is the reference. Moreover, a proportional–integral (PI) controller is used as a compensator, with the transfer function given as follows:

(12) $$K_{u}\left(s\right)=k_{c}\frac{\left(s+k_{i}\right)}{s}$$

where $k_{c}$ denotes the proportional gain and $k_{i}$ denotes the integral gain, the closed-loop of the DC voltage loop is given by the following:

(13) $$H_{cl}\left(s\right)=\frac{\frac{2k_{c}}{C_{dc}}\left(s+k_{i}\right)}{s^{2}+\frac{2k_{c}}{C_{dc}}+\frac{2k_{c}k_{i}}{C_{dc}}}$$

**Figure 3:**
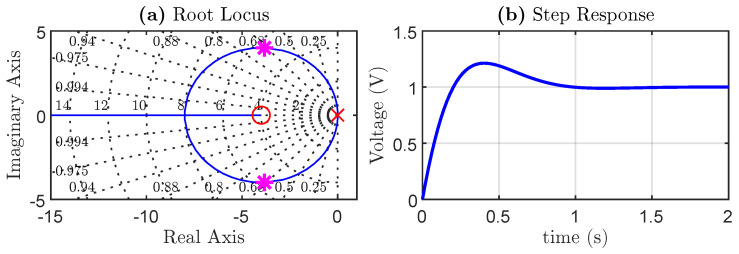
(**a**) Root locus and (**b**) step response of the DC controller.

In this work, a capacitance of 2.35 (mF) is considered, and the controller is tuned to achieve a settling time of 1 s and a damping constant of 0.707, as illustrated in [28]. The root locus and step response of the controller are shown in Figure 3. From Figure 3, it is evident that the system remains stable across the entire range of controller gains. Despite being designed with a 0.707 damping factor, the presence of a zero in the transfer function, with a real value closely aligned with that of the poles, leads to an overshot of around 20% for the highlighted closed-loop poles (depicted in pink). However, a 20% overshoot is deemed acceptable for the power converter, as in practical scenarios, the capacitor experiences minimal voltage fluctuations around the operating point. Consequently, the impact of the overshot on the power converter’s function is negligible.

## 3. Small-Signal Analysis of the Synchronverter

**Figure 4:**
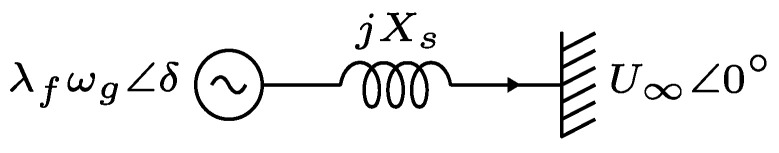
Small-signal model.

The synchronverter consists of a set of nonlinear equations. To simplify the analysis of the synchronverter, a reduced-order system is linearized. Two assumptions are made to reduce the order of the system. First, the resistance of the inductive filter can be considered negligible. Second, the dynamics of the AC output currents are neglected due to their fast response time, which is faster than the dynamics of the rotor [23]. Figure 4 shows the circuit of the new model.

The output functions are the active and reactive powers given by the following equation:

(14) $$y=h\left(x,u\right)=\left[\begin{array}{c} P_{g}\\ Q_{g} \end{array}\right]=\left[\begin{array}{c} \frac{\sqrt{\frac{3}{2}}\lambda_{f}\omega_{g}U_{\infty }\sin\;\delta }{X_{s}}\\ \frac{\sqrt{\frac{3}{2}}\lambda_{f}\omega_{g}U_{\infty }\cos\;\delta -U_{\infty }^{2}}{X_{s}} \end{array}\right]$$

where $X_{s}$ denotes the reactance calculated at nominal frequency. Replacing Equations (14) with Equation (2) and (7), the new model is as follows:

(15) $$\begin{array}{cc} \dot{x} & =f\left(x,u\right)=\left[\begin{array}{c} \dot{\omega_{g}}\\ \dot{\delta }\\ \dot{\lambda_{f}} \end{array}\right]=\left[\begin{array}{c} \frac{P_{g}^{*}}{\omega_{n}J_{g}}-\frac{\sqrt{\frac{3}{2}}\lambda_{f}U_{\infty }\sin\;\delta }{J_{g}X_{s}}+\frac{D_{p}}{J_{g}}\left(\omega_{n}-\omega_{g}\right)\\ \omega_{g}-\omega_{\infty }\\ \frac{Q_{g}^{*}}{K_{q}}+\frac{U_{\infty }^{2}-\sqrt{\frac{3}{2}}\lambda_{f}\omega_{g}U_{\infty }\cos\;\delta }{K_{q}X_{s}}+\frac{D_{q}}{K_{q}}\left(U_{\infty }^{*}-U_{\infty }\right) \end{array}\right] \end{array}$$

where $x={\left[\begin{array}{ccc} \omega_{g} & \delta & \lambda_{f} \end{array}\right]}^{T}$ denotes the state vector and $u={\left[\begin{array}{cccc} P_{g}^{*} & Q_{g}^{*} & U_{\infty } & \omega_{\infty } \end{array}\right]}^{T}$ denotes the input vector. It is worth mentioning that the first two inputs are controllable, and the two remaining inputs are non-controllable because they are perturbations.

The linear model is given by the following equations:

(16) $$\begin{array}{cc} \Delta \dot{x} & =A\Delta x+B\Delta u\\ \Delta y & =C\Delta x+D\Delta u \end{array}$$

where the arrays of this set of equations are presented in Appendix A. The transfer matrix of this system is as follows:

(17) $$G_{p}\left(s\right)=C{\left(sI-A\right)}^{-1}B+D$$

where $G_{p}\left(s\right)$ denotes the transfer function matrix and the output and input vectors in the Laplace domain are ΔY(s) and ΔU(s), respectively.

**Figure 5:**
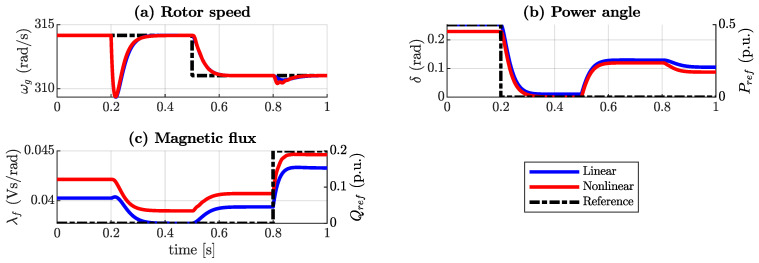
Simulation of the nonlinear fifth-order model and the reduced linear model, (**a**) rotor angular speed, (**b**) power angle, and (**c**) magnetic flux.

A simulation is performed to compare both the model in the previous section and the model given by (16). The results are shown in Figure 5. Initially, the system injected 50 W and 0 VAr into the grid. At t=0.2 s, −50 W of active power reference occurs. According to the figure, both models show that the rotor speed decreases but eventually returns to the nominal value. In addition, the power angle approaches 0 and the flux also decreases as the injected active power decreases to 0 W as expected. At t=0.5 s, the grid frequency decreases stepwise by 0.5 Hz, so the rotor speed decreases to the new synchronous speed. Due to the torque droop control of the synchronverter, the power angle increases in both models, indicating that active power is being injected into the grid. Finally, at t=0.8 s, 30 VAr of the reactive power reference is applied, so the magnetic flux increases. Both models show the same dynamics, but there is a difference between the steady-state values of the power angle and the magnetic flux, which is attributed to the resistance not considered in the linear model. This difference in values between the models is approximately less than 5%.

### 3.1. Active Power Loop

According to (17), the active power loop transfer function is defined as follows:

(18) $$\begin{array}{c} \Delta P_{g}\left(s\right)=G_{p_{11}}\left(s\right)\Delta P_{g}^{*}\left(s\right)+G_{p_{12}}\left(s\right)\Delta Q_{g}^{*}\left(s\right)+\ldots \\ G_{p_{13}}\left(s\right)\Delta U_{\infty }\left(s\right)+G_{p_{14}}\left(s\right)\Delta \omega_{\infty }\left(s\right) \end{array}$$

where $G_{p_{1j}}\left(s\right)$ denote the transfer functions of the first row of the transfer matrix in (17).

**Figure 6:**
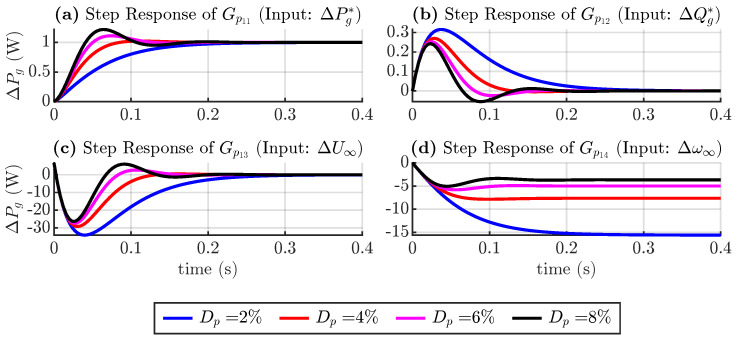
Step response of active power transfer functions considering torque droop coefficient variation.

In Figure 6, a step response is plotted for each transfer function of Equation (18), considering different values of the torque droop coefficient. These coefficients vary according to Equation (9), where the nominal apparent power and frequency are fixed. The inertia time constant is also fixed at 0.1 s. Figure 6a shows the real power output in response to a step change in the real power reference. As expected, a 1 unit change in the input causes a 1 unit change in the output. The lower frequency droop causes the system to be slower and overdamped. Figure 6b,c show the active powers, considering changes in the reactive power and voltage magnitude references. Changing these inputs does not change the steady state value of active power, but lower droop coefficients make the transient peak larger. Figure 6d shows the active power considering changes in the grid frequency, which reduces the active power as a percentage of the droop coefficient.

**Figure 7:**
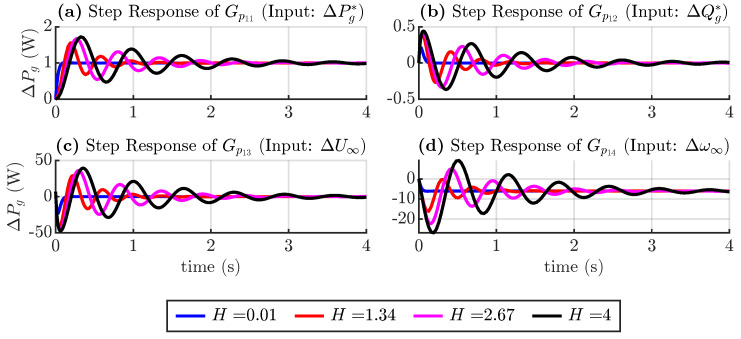
Step response of active power transfer functions considering inertia variation.

Similar plots are shown in Figure 7, but in this case, the torque droop coefficient is constant at 5%, and the inertia time constant changes from 0.01 to 4 s. The plots show that greater inertia results in a system with reduced damping and slower response. The steady-state values obtained are similar to the previous case.

### 3.2. Reactive Power Loop

**Figure 8:**
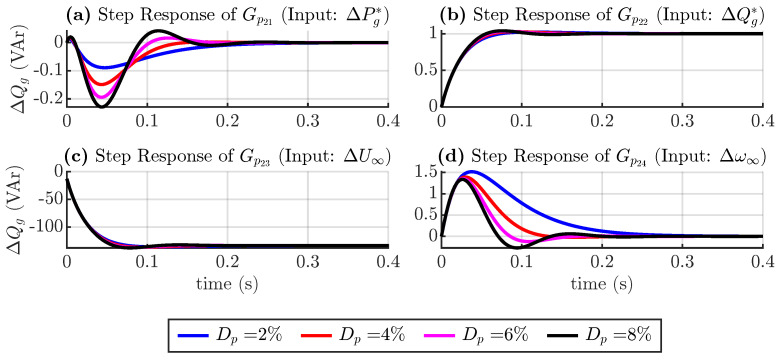
Step response of reactive power transfer functions considering torque droop coefficient variation.

**Figure 9:**
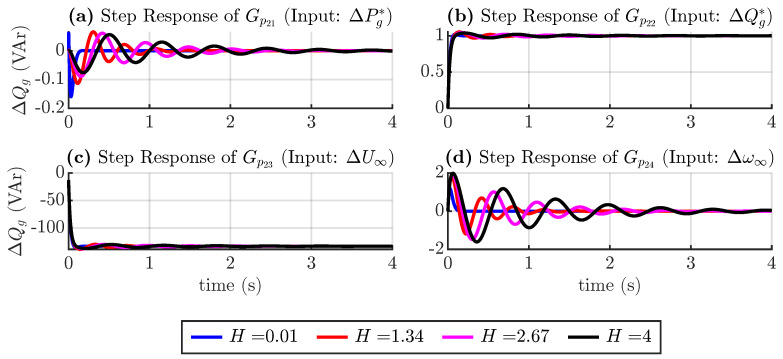
Step response of reactive power transfer functions considering inertia variation.

Similar to the active power loop, the reactive power loop transfer function is defined as follows:

(19) $$\begin{array}{c} \Delta Q_{g}\left(s\right)=G_{p_{21}}\left(s\right)\Delta P_{g}^{*}\left(s\right)+G_{p_{22}}\left(s\right)\Delta Q_{g}^{*}\left(s\right)+\ldots \\ G_{p_{23}}\left(s\right)\Delta U_{\infty }\left(s\right)+G_{p_{24}}\left(s\right)\Delta \omega_{\infty }\left(s\right) \end{array}$$

where $G_{p_{2j}}\left(s\right)$ denote transfer functions of the second row of the transfer matrix in (17). Figure 8 shows the response considering changes in the torque droop coefficient, and Figure 9 shows the response considering changes in inertia in Equation (19).

Figure 8a,d show that a change in the active power and frequency reference only produces a transient disturbance in the reactive power. Figure 8b,c show that a change in the torque droop coefficient produces a negligible change in the reactive power dynamics when the reactive power reference or voltage magnitude changes.

Figure 9 again shows that larger inertia produces a slower and less damped system.

### 3.3. Root Locus Analysis

**Figure 10:**
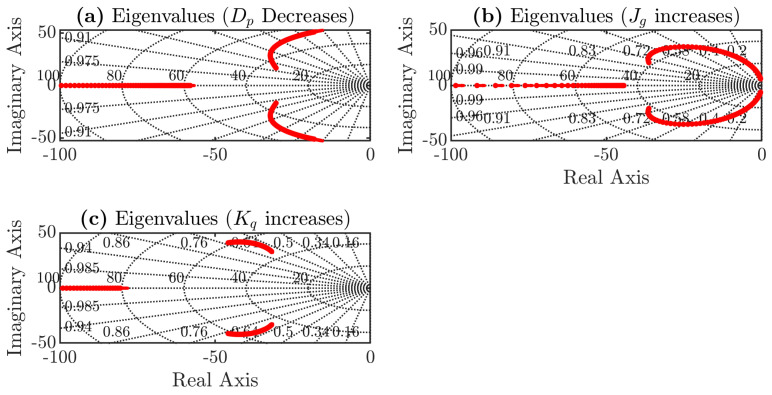
Root locus of synchronverter considering parameters variations, (**a**) torque droop coefficient varies, (**b**) inertia varies, and (**c**) gain $K_{q}$ varies.

Figure 10a shows the eigenvalues of the system, considering variations of the torque droop coefficient from 2% to 8%. There are two poles near the imaginary axis; these are the dominant poles of the system, demonstrating that the system behaves like a second-order system. As the percentage of the droop coefficient increases, the imaginary component of the dominant poles also increases, so this pole shift explains the overshoot obtained when the droop coefficient is larger.

Figure 10b shows the eigenvalues considering inertia variation, and similar to Figure 10a, there are two dominant poles. As the inertia increases, the imaginary part also increases and the poles move to the imaginary axis. At some point, the imaginary part stops increasing and decreases, while the poles move closer to the origin. This shift explains the increase in settling time and the oscillatory component of the step response in the previous section.

Finally, Figure 10c shows that increasing the gain Kq makes the system less damped and has less impact on the settling time of the system compared to the other two parameters.

## 4. Maximum Power Point Tracking Method

The DC source of the inverter in this paper is a PV array, which implies the need for a DC voltage compensator to control the injected power from the array. Also, this source requires a maximum power point tracking (MPPT) algorithm to obtain the voltage that allows extracting the maximum power, as this voltage is variable depending on the environmental conditions, such as irradiance and temperature.

### 4.1. Solar Cell Model

**Figure 11:**
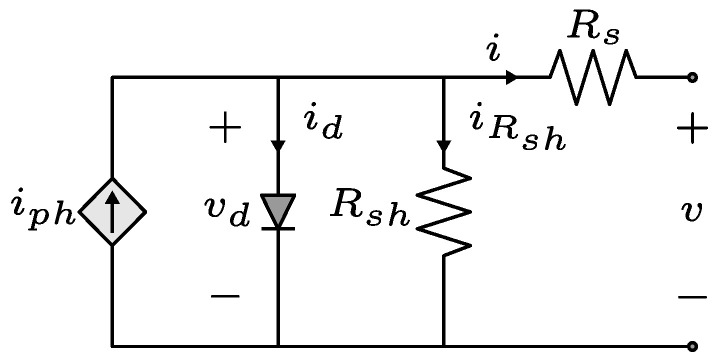
Single diode solar cell model.

The solar cell model is shown in Figure 11. The current source $i_{ph}$ represents the photocurrent, which depends on the irradiance. The diode in antiparallel represents the P-N junction of the semiconductor and the resistors $R_{s}$ and $R_{sh}$ represent the internal losses of the cell [29].

By applying the current Kirchhoff law in this circuit, the expression of the output current *i* is as follows:

(20) $$i=i_{ph}-i_{d}-i_{R_{sh}}=i_{ph}-i_{0}\left(e^{\frac{qv_{d}}{nkT}}-1\right)-\frac{v+iR_{s}}{R_{sh}}$$

where $i_{d}$ denotes the diode current, $i_{R_{sh}}$ denotes the current of the shunt resistor, $i_{0}$ denotes the inverse saturation current, $v_{d}$ denotes the diode voltage, *k* denotes the Boltzmann constant, *T* denotes the absolute temperature, *n* denotes the ideality diode factor, and *q* denotes the electron charge.

**Figure 12:**
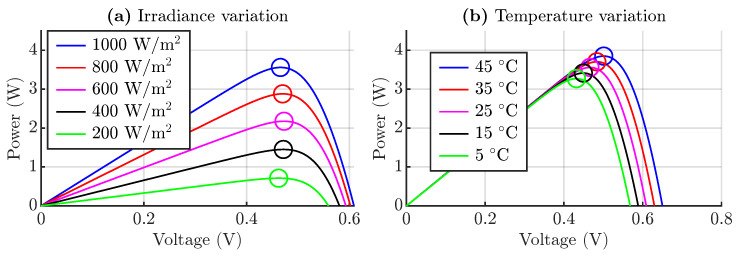
Solar cell curves, (**a**) power of the solar cell considering irradiance variation and (**b**) power of the solar cell considering temperature variation.

As shown in Figure 12a, the irradiance has a direct relationship with the power available from the cell. Figure 12b shows that the temperature has less influence on the amount of power available in the cell, but it significantly changes the voltage of the maximum power point marked by a circle.

### 4.2. MPPT with Measurements Cells

**Figure 13:**
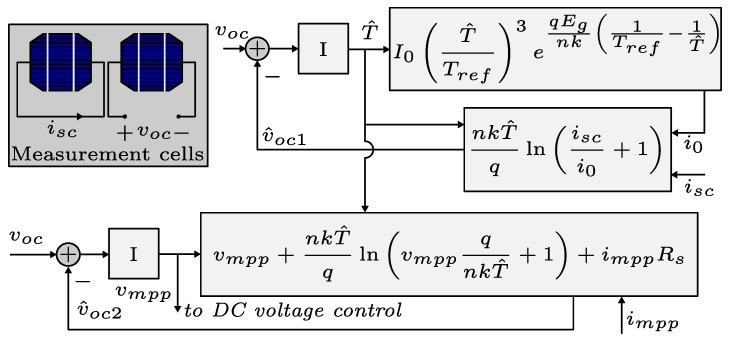
MPPT based on measurement cells.

In this paper, an MPPT based on measurement cells is used [30]. These cells measure the open-circuit voltage and short-circuit current. The measurements are sent to the control loops shown in Figure 13, which allows us to obtain the maximum power point (MPP) voltage.

In order for the synchronverter to provide frequency support via the droop coefficient, it is critical to determine both the maximum available power and the required injected power according to the droop function. Since the PV array power depends on the DC bus voltage, a new voltage reference is required to inject this power. The first step is to define the expression for estimating the available power, which is given by the following:

(21) $$p\approx i_{sc}v-i_{0}\left(e^{\frac{qv_{d}}{nkT}}-1\right)v$$

where this expression neglects the resistances of the cell model. To inject power lower than the maximum, it is necessary to find a new expression that allows obtaining this new operating voltage. The curves of Figure 12 show an approximately linear relationship between the power and cell voltage on the left side of the MPP; therefore, the new operating voltage could be estimated by the following:

(22) $$v_{op}\approx v_{mpp}+\frac{P_{op}-P_{mpp}}{\frac{dp}{dv}}$$

where $P_{op}$ denotes the new power injected into the grid, $P_{mpp}$ denotes the power of the MPP and could be calculated using the MPP voltage in (21), and dp/dv denotes the slope on the left side of the cell power curve. The power $P_{op}$ is calculated using the following equation:

(23) $$P_{op}=P_{mpp}+\omega_{n}D_{p}\left(\omega_{n}-\omega_{\infty }\right)$$

where the frequency $\omega_{\infty }$ is obtained from the PLL.

## 5. Results

**Figure 14:**
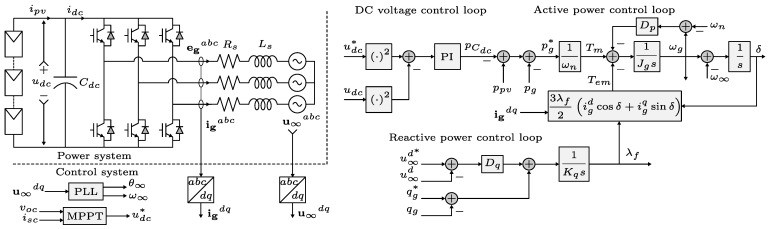
Proposed strategy control and power system.

The complete control strategy is shown in Figure 14. The system consists of an inverter connected to an infinite busbar via an inductive filter. The DC source is a PV array. The voltage and current are measured and transformed into a dq frame. The DC voltage reference is obtained from the MPPT. This reference could be used if it is needed to inject the maximum power, otherwise, a new voltage reference could be used. The DC voltage loop calculates the active power reference to send to the active power loop of the synchronverter. The reactive power loop of the synchronverter could be adjusted to compensate for voltage or reactive power. The proposed control strategy is validated by the simulation and experimental setup.

### 5.1. Simulation Results

To validate the proposed strategy, the system shown in Figure 14 was simulated in the software PSIM^®^ 2022, Altair Engineering 1820 E. Big Beaver Rd., Troy, MI, USA, considering the parameters of Table 1. The rating power, voltage, and frequency of the system are 3 kVA, 380 V, and 50 Hz, respectively.

**Figure 15:**
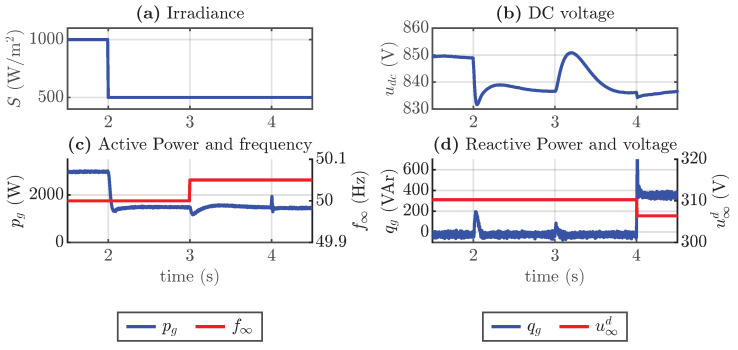
Simulation results, (**a**) irradiance step change, (**b**) active power and frequency, (**c**) reactive power and grid voltage magnitude, and (**d**) DC voltage.

The simulation considers an initial condition of nominal power injection of 3 kW from a PV array of 50 modules, each rated at 60 W in a series connection. The initial reactive power injected into the infinite bus bar is 0. At t=2 s, an irradiance step reduction occurs as shown in Figure 15a; thus, it can be seen from Figure 15b that the DC voltage is reduced from approximately 849 V to 836 V, given that the DC voltage reference is modified by the MPPT. This reduction in irradiance also implies a reduction in active power as shown in Figure 15c. At t=3 s, there is a step increase in the frequency of 0.05 Hz. Figure 15b shows that the DC voltage increases to 850 V and then returns to the previous value. This is because the torque droop coefficient reduces the power injection, so the energy injected from the PV array is stored in the capacitor until the energy is returned to the grid because the DC voltage controller reduces the voltage to reach the reference. At t=4 s, the busbar voltage magnitude decreases from 310.5 V to 306.3 V. This change results in an increase of 309.5 VAr of reactive power injection due to the effect of the reactive power droop coefficient, as shown in Figure 15d.

**Figure 16:**
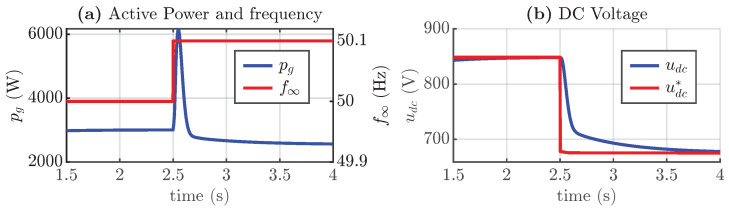
Simulation of synchronverter including frequency droop correction, (**a**) active power and frequency change, and (**b**) DC voltage and voltage reference.

As shown in the previous simulation, changing the frequency of the grid in a steady state did not result in a change in active power injection, despite the use of droop control. This is due to the fact that the power injection from the array is DC voltage-dependent. Therefore, to change the power injection, the DC voltage reference needs to be adjusted. To do this, the estimation of the new voltage operation in the MPPT section is implemented. The simulation results are shown in Figure 16, where the frequency change causes a change in the voltage reference and, therefore, in the active power. This disturbance and the change in the voltage reference produce a noticeable peak transient in the active power.

### 5.2. Experimental Results

**Figure 17:**
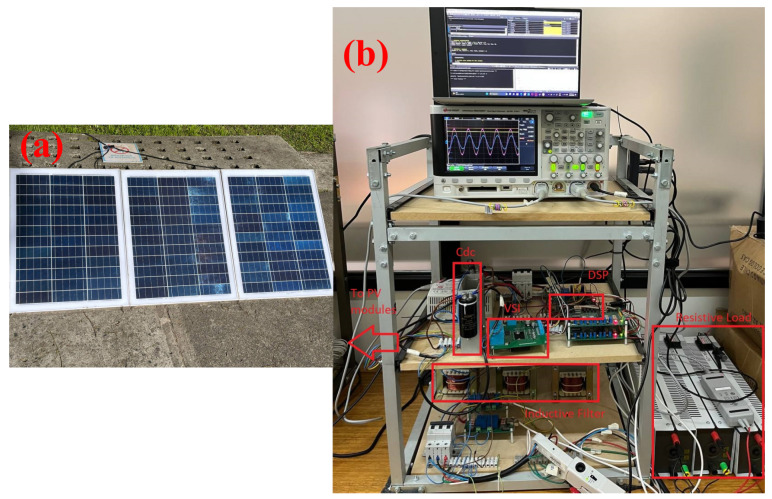
(**a**) PV modules and (**b**) experimental setup.

This control strategy was implemented using an insulated gate bipolar transistor (IGBT) power converter connected to a California Instruments CSW 5550 variable AC power source through an inductive filter. A resistive load was connected between the inverter and the AC source since the source was not regenerative. The DC source was a PV array consisting of 3 ESUN modules of 50 W, each connected in series. A Texas Instruments DSC TMS320F28335 microcontroller was used and the computation burden was 55 μs, which was close to computing a model-based predictive control, as shown in [31]. The commutation frequency was set to 10 kHz. The variables were measured using a Keysight MSOX3054T 4-channel oscilloscope. The setup is shown in Figure 17 and the parameters of the setup are shown in Table 2.

**Figure 18:**
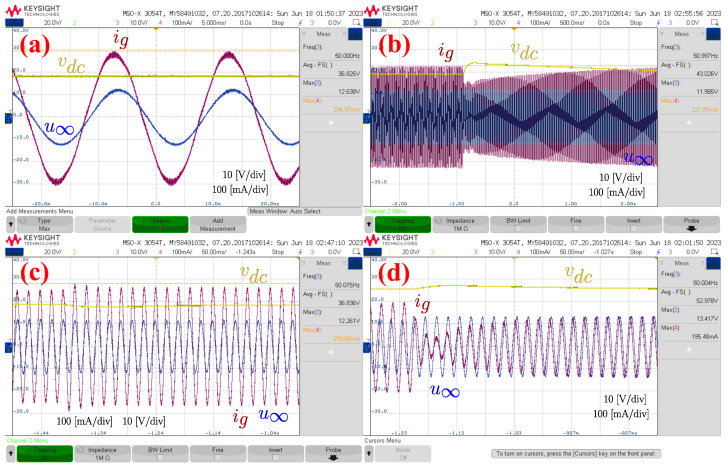
Experimental results, (**a**) synchronverter injecting PV power, (**b**) frequency increment of 1 Hz, (**c**) sag of 5%, and (**d**) swell of 5%.

The experiment consisted of feeding PV power from the array through the test setup’s synchronizer while disturbances occurred. Initially, the synchronizer supplied 50 W of active power and 0 VAr of reactive power. To achieve this, the DC voltage was adjusted to 36.8 V; see Figure 18a. The DC voltage is in yellow, the grid voltage of phase a is in blue, and the current of phase a is in red. Under these conditions, the peak AC voltage is 12.6 V and the peak current is 2.9 A. In Figure 18b, an increment of 1 Hz is applied to the AC source. Similar to the simulations, this increment produces an increment in the DC voltage, but the DC voltage control returns the voltage to the reference of 36.8 V. It should be noted that the droop correction implemented in the simulation was not implemented in the experimental setup.

Figure 18c shows the variables when a 5% sag occurs. In this case, the reactive power injection is increased to 50 VAr because the reactive power droop coefficient was adjusted to 10%. This change is stabilized after about 100 ms because the inertia time constant was set to 0.1 s. The droop also causes a transient decrease in the DC voltage, but it is restored after 1 s due to the action of the DC voltage regulator.

Finally, Figure 18d shows the variables when a 5% swell occurs. Initially, the synchronverter was injecting 0 VAr because the reference was fixed to that value. The transient causes a change of −50 VAr in the injected reactive power. Because of this change, the peak current is reduced and the current now leads the voltage. With respect to the DC voltage, the voltage increases for 50 ms and then decreases to the reference value.

### 5.3. Comparison with Other Works

Table 3 compares the proposed control with other solutions that address the frequency and power compensation problems or voltage compensation. The functions included in the table are the ability to compensate voltage, specifically to maintain voltage magnitude within safe levels; active and reactive power injections in response to grid needs; frequency support, which involves adapting the behavior of the device to improve or maintain system stability in terms of frequency; and energy storage capacity.

## 6. Conclusions

This paper presents the implementation of a PV synchronverter control strategy, employing a virtual synchronous machine approach. Through simulations and experimental setups, this strategy facilitates voltage and frequency support functions, along with active power injection from a PV inverter. The incorporation of inertia is facilitated by the developed control algorithm, enabling the power converter to emulate the behavior of a synchronous machine, thereby providing support to the grid when necessary. This concept is becoming increasingly imperative as PV solar systems proliferate within power generation systems. Without intervention, the interconnected systems face a heightened risk of instability, amplifying the likelihood of widespread failures. The results demonstrate the feasibility of injecting PV power while controlling the inverter as a synchronverter. This synchronverter effectively compensates reactive power during voltage sag and swell events, ensuring the seamless injection of PV power without compromising synchronization with the grid. Reactive power plays a crucial role in voltage amplitude regulation and can contribute to enhancing the stability of distribution grids. However, at the initial stage of introducing the proposed control method, the authors focused on considering an infinite bus scenario, which reflects the reality for most distribution lines connected to robust interconnected systems. However, this concept is less applicable when dealing with a more detailed model of a distribution system or microgrids. Therefore, future work will consider a non-infinite bar where the changes in voltage magnitude produced by the compensation will be observed. Frequency support requires the ability to absorb/supply power changes from the grid, and it is necessary to maintain energy reserves that are traditionally found in the inertia of synchronous machines or batteries. Since the PV synchronverter does not have an energy storage system, the injection of solar energy must be reduced, for which it will be necessary to implement optimization algorithms to maintain a sufficient energy margin, provide frequency support, and not significantly damage the economic benefits of the PV generator. Also, the transient response of the active power loop must be improved since, as seen in the simulations, there are power peaks that can cause damage to the grid. Nevertheless, an MPPT algorithm is utilized to ensure proximity to the maximum power point (MPP), optimizing the injected energy. This algorithm leverages a measuring cell approach, offering the dual benefits of ease of implementation and low computational overhead. Importantly, the overall computational burden of the algorithm remains modest, enabling implementation on cost-effective digital boards, such as the ones referenced in the experimental results. Consequently, this manuscript not only contributes to system stability but also mitigates the need for costly energy storage systems, requiring only minimal additional code lines in the control script.

## Figures and Tables

**Table 1:** Simulation parameters.

Parameter	Value	Parameter	Value
$S_{\mathrm{nom}}$	3000 VA	$V_{\mathrm{nom}}$	380 V_RMS_
$f_{\mathrm{nom}}$	50 Hz	$Z_{\mathrm{nom}}$	48.13 Ω
$R_{s}$	1 Ω	$L_{s}$	10 mH
$P_{pv}$	60 W	$C_{dc}$	2.35 mF
PV modules	50	*H*	0.4 s
PV strings	1	$D_{p}$	3.039 Nms/rad
PV $v_{oc}$	21.1 V	$D_{q}$	96.77 VAr/V
PV $i_{sc}$	3.8 A	$K_{q}$	1000 VAr/V
PV $v_{mpp}$	17.1 V	$k_{c}$	0.009
PV $i_{mpp}$	3.5 A	$k_{i}$	4
$f_{sw}$	20 kHz		

**Table 2:** Experimental setup parameters.

Inverter Parameters	Value	PV Parameters	Value
$S_{\mathrm{nom}}$	100 VA	$V_{\mathrm{nom}}$	15 V_RMS_
Module number	3	$v_{oc}$	21.71 V
$R_{s}$	0.25 Ω	$i_{sc}$	2.92 A
$L_{s}$	3.7 mH	$v_{mpp}$	18.2 V
$C_{dc}$	2.35 mF	$i_{mpp}$	2.78 A

**Table 3:** Comparison.

	This Paper	PV-STATCOM [17]	VSM 2-Stages [26]	Batteries [19]
Voltage compensation	✓	✓	✓	-
Power reactive compensation	✓	✓	✓	-
Active power injection	✓	✓	✓	✓
Frequency support	✓	-	✓	✓
Energy storage capability	-	-	-	✓

## Data Availability

The data presented in this study are available on request from the corresponding author.

## Abbreviations

The following abbreviations are used in this manuscript:

PV	photovoltaic
DC	direct current
FACTS	flexible AC transmission system
MPPT	maximum power point tracking

## Appendix A

Arrays of the linearized system.

$$\begin{array}{cc} A & =\left[\begin{array}{ccc} -\frac{D_{p}}{J_{g}} & -\frac{\sqrt{\frac{3}{2}}\lambda_{f}^{o}U_{\infty }^{o}\cos\;\delta^{o}}{X_{s}J_{g}} & -\frac{\sqrt{\frac{3}{2}}U_{\infty }^{o}\sin\;\delta^{o}}{X_{s}J_{g}}\\ 1 & 0 & 0\\ -\frac{\sqrt{\frac{3}{2}}\lambda_{f}^{o}U_{\infty }^{o}\cos\;\delta^{o}}{X_{s}K_{q}} & \frac{\sqrt{\frac{3}{2}}\omega_{g}^{o}\lambda_{f}^{o}U_{\infty }^{o}\sin\;\delta^{o}}{X_{s}K_{q}} & -\frac{\sqrt{\frac{3}{2}}\omega_{g}^{o}U_{\infty }^{o}\cos\;\delta^{o}}{X_{s}K_{q}} \end{array}\right]\\ B & =\left[\begin{array}{cccc} \frac{1}{\omega_{n}J_{g}} & 0 & -\frac{\sqrt{\frac{3}{2}}\lambda_{f}^{o}\sin\;\delta^{o}}{X_{s}J_{g}} & 0\\ 0 & 0 & 0 & -1\\ 0 & \frac{1}{K_{q}} & \frac{2U_{\infty }^{o}-D_{q}X_{s}-\sqrt{\frac{3}{2}}\omega_{g}^{o}\lambda_{f}^{o}\cos\;\delta^{o}}{X_{s}K_{q}} & 0 \end{array}\right]\\ C & =\frac{\sqrt{\frac{3}{2}}U_{\infty }^{o}}{X_{s}}\left[\begin{array}{ccc} \lambda_{f}^{o}\sin\;\delta^{o} & \omega_{g}^{o}\lambda_{f}^{o}\cos\;\delta^{o} & \omega_{g}^{o}\sin\;\delta^{o}\\ \omega_{g}^{o}\cos\;\delta^{o} & -\omega_{g}^{o}\lambda_{f}^{o}\sin\;\delta^{o} & \omega_{g}^{o}\cos\;\delta^{o} \end{array}\right]\\ D & =\frac{1}{X_{s}}\left[\begin{array}{cccc} 0 & 0 & \sqrt{\frac{3}{2}}\omega_{g}^{o}\lambda_{f}^{o}\sin\;\delta^{o} & 0\\ 0 & 0 & \sqrt{\frac{3}{2}}\omega_{g}^{o}\lambda_{f}^{o}\cos\;\delta^{o}-2U_{\infty }^{o} & 0 \end{array}\right] \end{array}$$
